# Sleep Quality and Its Correlates among Adolescents of Western Nepal: A Population-Based Study

**DOI:** 10.1155/2021/5590715

**Published:** 2021-05-16

**Authors:** Prayas Gautam, Maginsh Dahal, Kushalata Baral, Rohit Acharya, Sudip Khanal, Aastha Kasaju, Raj Kumar Sangroula, Koshish Raj Gautam, Kabita Pathak, Anu Neupane

**Affiliations:** ^1^School of Public Health, Chitwan Medical College, Chitwan, Nepal; ^2^School of Public Health, Nanjing Medical University, Nanjing, China; ^3^Department of Public Health, Nobel College, Pokhara University, Sinamangal, Kathmandu, Nepal; ^4^Population Services International/Nepal, Western Regional Hub, Nepalgunj, Nepal; ^5^Department of Public Health, Little Buddha College of Health Science, Minbhavan, Kathmandu, Nepal; ^6^Nitte University, Karnataka Mangaluru, India; ^7^Department of Humanities, Pashupati Multiple Campus, Tribhuvan University, Chabahil, Kathmandu, Nepal

## Abstract

Sleep quality has a long-term impact on health leading to depression among adolescent students. We conducted a cross-sectional study to assess the prevalence of sleep quality and its associated factors among adolescents of western, Nepal. 514 adolescents from different schools were selected by the probability proportionate to size (PPS) method. The Pittsburgh Sleep Quality Index (PSQI) was used to assess the sleep quality among adolescents. The collected data were entered in EpiData 3.2 version, then extracted to excel 2019 and was analyzed with the help of RStudio (version 1.2.5033). Frequency distribution and percentage were identified as descriptive analysis whereas chi-square test was done. Variables that were found statistically significant (*P* < 0.05) were further analyzed using the logistic regression model. The prevalence of sleep quality in this study was 39.1%. In a bivariate analysis, ethnicity, religion, place of residence, drinking status of father, reason for selecting the currently studying faculty, satisfaction with academic performance, use of tobacco, relationship with friends or classmates, more use of internet per day, and use of internet before falling asleep were found to be statistically significant with sleep quality. Those students who left their home without informing their parents were more than three times at the risk of sleep quality than those students who never ran away from their home without informing their parents (AOR = 3.435, CI: 1.237-9.540). The overall prevalence of sleep quality among school going adolescent students was 39.1 percent which was comparatively high.

## 1. Introduction

Sleep is an important physiological process for human beings. It is considered one of the major contributing factors for the physical and mental health well-being, especially among the adolescents. Sleep plays (vital/essential roles in the) somatic, cognitive, and psychological process [1]. Even though the direct benefits of sleep is not well quantified across many populations, it is understood that sleep disorder has serious health issues [2]. Sleep deprivation is the condition of not having enough sleep than the average [3]. The amount of sleep required may vary from person to person but on average most of the adults required 8-10 hours of sleep from age 14 to 17 years and 7-9 hours of sleep from age 18 to 25 years [4]. Sleep deprivation can be either chronic or acute.

Adolescents with sleep deprivation report more depression, anxiety, inattention, conduct problem(s), drug and alcohol use (abuse), impaired academic performance, and suicidal thoughts and behaviors [3, 5]. Sleep habits include bedtime, wake-up time, and sleep duration [6]. There are numerous evidences regarding the negative effects of sleeping disorders on mental health of adolescents [7, 8]. The available research advocates that sleeping disorders are related with shortfall in functioning across a wide range of indicators of psychological, interpersonal, and well-being [9]. Sleep disorders are considered to be harmful to adolescents as it may decrease the work efficiency and learning ability [10]. Sleep quality leads to issues with learning and behaviors. Adolescents who do not get proper and adequate sleep are more likely to (be) inattentive, distracted, uninterested, impulsive, and hyperactive [11]. Sustained, untreated sleeping disorder may lead to major depression, anxiety disorders, and substance abuse [12, 13].

Various factors decide sleep quality (such as age, gender, habitat, BMI, physical activity or sports) [14]. Heavy smoking, frequent alcohol and coffee intake, lack of regular exercise, poor diet, and skipping breakfast are associated with short sleep duration and insomnia among adolescents. Short-term effects of sleep disorder in school-aged children and adolescents manifest as daytime fatigue only while medium-term effects have been associated with daytime sleepiness and behavior problems. Attention deficit/hyperactivity disorder has been associated with sleep disorders among children and adolescents [3, 5]. Evidences show a strong association between sleep quality and poor academic performance of the adolescents [10].. Epidemiological studies conducted in western Europe, the USA, and Japan have reported a prevalence of sleeping disorder-related symptoms ranging from 20% to 48% [15]. Students experiencing number of sleep problems may have impact on their academic performance, health, and mood. Sleep quality is the common problem among college students. Both biological and social factors contribute to sleep quality [16]. Adolescents' sleep pattern requires specific attention because it may affect their academic environment. Adolescents comparatively have insufficient sleep than younger children because of their daily schedule and the physical, mental, and emotional changes they are going through [15]. Thus, we tried to assess sleep quality and its correlates among adolescents of western Nepal.

## 2. Methods

A school-based descriptive cross-sectional study was conducted among the adolescent students of age 15-19 years currently studying in grade 11 and 12 of western rural Nepal.

### 2.1. Sample Size

The sample size was determined by using the prevalence of 21.2% (prevalence was obtained from study conducted in Nepal among undergraduate students) [17] with confidence level 95% and allowable error 5%.

We used the following formula to calculate the sample size:

(1) $$\begin{array}{c} n_{0}={\left(\frac{Z_{\alpha }}{E}\right)}^{2}PQ, \end{array}$$

where *n* is the required sample size, *α* = 5% (desired level of significance), *Z*_*α*_ = *Z*_0.05_ = 1.96 (from normal table), *P* = 21.2%, i.e., 0.212 (prevalence), *Q* = 1 − *P* = 0.788, and *E* = 0.05 (desired error).

(2) $$\begin{array}{c} n_{0}={\left(\frac{1.96}{0.05}\right)}^{2}\times 0.212\times 0.788=256.7\sim 257. \end{array}$$

Adjusting the design effect the sample size *n* = 257 × 2 = 514.

### 2.2. Sampling Technique

Among the total schools, 16 were selected randomly by using the lottery method. Among them, 8 were private schools and 8 were public schools. The total number of students in public was 3879 and private was 3524. After that by using probability proportionate to size (PPS), schools were selected in equal number, i.e., four from each category. As the total number of students in both public and private were almost equal. Then, in the second stage, the sample size from each school was determined proportionately, and finally, further stratification was done in class 11 and 12 and from each faculty (science, management, and education). The sample frame was prepared from the school attendance. The required sample of 514 was selected by a random number table through excel. Adolescent students aged between 15 and 19 years who were present during the day of data collection were included in this study. Those students who were absent and refused to participate were replaced by randomly selected new participants.

### 2.3. Data Collection Tools

Semistructured, self-administrated questionnaire was used for data collection. The questionnaire included sociodemographic information and behavioral and psychological characteristics of students. Sleep quality was measured by the Pittsburgh Sleep Quality Index (PSQI). PSQI is an effective instrument used to measure the quality and patterns of sleep. It is a brief, reliable, standardize valid self-report instrument. It differentiates “poor” from “good” sleep by measuring seven domains: subjective sleep quality, sleep duration, sleep latency, habitual sleep efficiency, sleep disturbances, use of sleep medication, and daytime dysfunction over the last month. Scoring of the answer was based on a “0” to “3” scale, whereby 3 reflected the negative extreme on Likert's scale. PSQI score ranges from 0 to 21 in which a greater score suggests poor sleep quality. PSQI global score > 5 was used which had a sensitivity of 89.6% and specificity of 86.5%, to determine the quality of sleep of adolescent students In this study, only a self-rated questionnaire are included [18].

### 2.4. Data Collection Procedure and Technique

The questionnaire was in Nepali language and piloted in 50 individuals before use in the survey. A translated version of PSQI used in this study has Cronbach's alpha of 0.76. Participants were briefed about the study objectives, and parental consent was taken for the respondents aged below 18 years. Data were collected in separate classroom(s). Participants were briefed about the techniques of filling the questionnaire. Seating arrangements of the students were made properly in such a way that chances of peeking each other's answers were as low as possible.

### 2.5. Statistical Analysis

Data were coded, edited, entered, and rechecked by a researcher. The collected data were entered in EpiData 3.2 version and then extracted to excel 2019. The final data was analyzed with the help of RStudio (version 1.2.5033).

The data analysis was carried out using descriptive statistics and inferential statistics. Univariate analysis was presented using frequency distribution and pie charts. Bivariate statistics such as chi-square tests and *t*-tests were computed to test the differences in sociodemographic characteristics among the adolescents. Multivariate analysis was carried out using logistic regression using the following formula [19]:

(3) $$\begin{array}{c} \ln\left(\frac{p}{1-p}\right)=\alpha +\beta_{1}x_{1}+\beta_{2}x_{2}\cdots \cdots ..+\beta_{p}x_{p},\\ p_{\left(y/x\right)}=\frac{\exp\left(\alpha +\beta_{1}x_{1}+\beta_{2}x_{2}\cdots \cdots ..+\beta_{p}x_{p}\right)}{1+\exp\left(\alpha +\beta_{1}x_{1}+\beta_{2}x_{2}\cdots \cdots ..+\beta_{p}x_{p}\right)}, \end{array}$$

where odds ratio is OR = exp^*β*^ and confidence interval is $\exp\left[\hat{\beta }\pm Z_{1-\alpha /2}\times \hat{\text{SE}}\left(\hat{\beta }\right)\right]$.

## 3. Results

Table 1 represents the demographical and economical characteristics of 514 respondents. Mean age (in years) of the respondents was 17.4 (SD ± 0.92). Almost half of the students were male (52.5%). Almost three-fifths of the participants (59%) were from the advantaged caste group (Brahmin/Chhetri), whereas 89.7% of the students were from Hindu religion, and almost four-fifths (78%) were receding in the urban area. Majority (65.8%) of the students live in a nuclear family and family size as mean ± SD of 5.51 ± 2.12. Majority (91.6%) of the parents were living together. More than 41% of the family monthly income was more than twenty-five thousand (Nepalese rupees), which is considered a middle income in Nepalese context. Regarding characteristics of respondent's parents, about 69% of the respondent's father had studied below secondary level and more than eight out of ten (82%) of the respondents' mother had studied below secondary level. Most of the student's parents were involved in agriculture (53.5%) as their major occupation. Almost two-fifths (37.9%) of the respondent's fathers consumes alcohol but majority (95.1%) of the respondent's mother never had alcohol.

**Figure 1:**
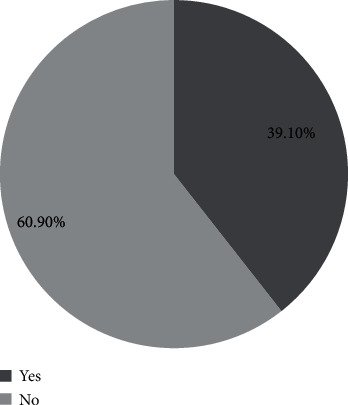
Prevalence of sleep quality using the Pittsburgh Sleep Quality Index (PSQI) (*n* = 514).

Figure 1 shows that the prevalence of sleep quality was found to be 39.1% among adolescents.

Table 2 represents educational characteristics of the students. 55.8 percent of the respondent was from grade 11. More than forty out of hundred (41.1%) were studying in the management faculty. Four-fifths (80.4%) of the students currently studying their respective faculty was because of their own preference and more than three-quarters (78.2%) passed in their previous exams. About two-thirds (64.6%) of the students were not satisfied with their academic performance. It also represents the behavioral characteristics of respondents. Majority (93.2%) of the students were nontobacco users, and 93.8% of students has never had alcohol. Almost half of the respondents used internet up to two hours daily. About 67% of the respondents used minimum of 1 hour of internet before going to bed. Similarly, it portrays psychological factors of respondents. More than three-quarters (79.0%) report that they have had conflict in their families. Majority (93.0%) of the parents never scold their children more often. Almost half (49.2%) of the students had a good relationship with friends, and about one-third (32.3%) of students had good relationship with teachers as well. About one-third (29.4%) of the respondents felt lonely. Less than one-tenth (8.0%) of the students had left their home ever in their life without informing their parents regardless to the frequency of running away from home. More than one-fifth (23.2%) of the students had tried to hurt themselves irrespective to the frequency of hurting themselves. Students frequently shared their feeling more with their close friends than with family.

Table 3 represents the bivariate and multivariate analyses of factors associated with sleep quality. Those students who were greater than 17 years, 1.55 times (AOR = 1.55, CI: 1.03-2.33) more likely at the risk of sleeping quality compare to those students whose age were less than equal to 17 years. Moreover, the selection of their current study by others was 1.77 (AOR = 1.77, CI: 1.05-3.00) times more likely at the risk of sleeping quality compared to those who decides him/herself for their study. The poor relation with friends was 2.12 (AOR = 1.12, CI: 1.13-4.00) times more likely at risk of sleeping quality compared to the relationship that was good with their friends. Those students who left their home without informing their parents were more than three times at the risk of sleep quality than those students who never ran away from their home without informing their parents (AOR = 3.15, CI: 1.44-7.21). Furthermore, students spending more than one hour before going to bed was 2.67 (AOR = 2.67, CI: 1.61-4.48) times more likely at the risk of sleep quality than those students that use internet less than or equal to one hour.

## 4. Discussion

This was a correlation study with a primary purpose of finding the factors associated with sleep quality among adolescents of western Nepal.

In this study, the percentage of boys and girls was almost equal. All the school students were aged from15 to 19 years. 58.8 percent of the respondents were from Brahmin or Chhetri, and 89.7 percent of the students followed Hinduism. Mean family size was 5.51 (±2.12SD) which was higher than National 4.6 [20]. Almost half of the students' fathers were engaged in agriculture, and 56.6 percent of student's mothers were engaged in agriculture. Majority of the students were nonalcoholic and nontobacco users. Three-quarters of the students had smart phones, and 87.6 percent of the students had access to the internet.

### 4.1. Prevalance of Sleep Quality

In this study, the prevalence of sleep quality was found to be 39.1 percent. Our results indicate that sleep quality was not rare among adolescent students of Dang district. This is similar to the research conducted among Chinese adolescent students by Gou et al. in 2014 [21]. Similar prevalence was observed in a study conducted in Gwalior, India, where the prevalence was 37.6 percent [22] and a study conducted in Thailand where the prevalence was 42.4 percent [23]. A study was conducted in Thailand by Seblew et al. where prevalence was 55.8 percent which is comparatively higher than our study findings. This might be due to the age of students being up to age 25 and sample size was comparatively higher (2551) than our study [2]. Prevalence was less (24.0%) compared to our in a study conducted by Kesintha et al. in Malaysia among school adolescents. The differences might be due to the research conducted in different countries with different settings, and the sample size was comparatively higher than our study [24]. A study conducted by Bhandari et al. among undergraduates of Nepal displayed almost similar prevalence (35.4%) of sleep quality [17].

### 4.2. Demography and Sleep Quality

Demographic factors such as sex, age, family type, family size, monthly income, parents' marital status, education of parents, and employment of parents were not significantly associated with sleep quality. A study conducted in Ethiopia and India had also found similar findings [2, 22]. A study conducted in Southern Thailand has prevalence of male and female (42.6% and 42.3%) which is similar to our findings (38.1% and 40.1%) [23]. Monthly income was also found to be insignificant to sleep quality in a research conducted in Malaysia, but in similar research, father's and mother's education levels were found to be significant predictors of sleep quality which was different than in our findings [24]. In a study conducted among Chinese adolescent students, gender and family economic status were significantly associated with sleep quality which is different than our findings [21]. The nonsignificant association of income with sleep quality in this study was may be due to majority of the students being unsure about the exact family income. In this study, ethnicity, religion, and place of residence of students were found to be statistically significant. Non-Brahmin/Chhetri was almost two times more likely to have sleep quality than the students from Brahmin/Chhetri ethnicity (OR = 1.44, CI: 1.01-2.07), whereas a study conducted by Bhandari et al. showed no association between ethnicity and sleep quality among students [17]. Similarly, religion was found to be statistically significant where non-Hindu people were two times more likely to have sleep quality compared to Hindu students (OR = 2.11, CI: 1.19-3.79), whereas in a study conducted in Nepal, non-Hindu students had better sleep quality. Permanent place of residence was significantly associated with sleep quality where rural people suffer more sleep quality but the study conducted in Egypt urban students were more to have poor sleep compared to rural. This might be due to different study settings [25].

From our study, students studying in different grades, faculty (science, management and education), and achievement in previous exams were found to be insignificant with sleep quality. However, in a study conducted by Ismail et al., type of school (public and private) was found significantly associated with sleep quality where poor sleepers were significantly higher among public students compared to private [25]. Bhandari et al. in a study conducted among undergraduate students found a significant association between achievements in previous exams and sleep quality which was different from our findings [17]. Grade was also insignificant in this study, but Saxena et al. in a cross-sectional study in Gwalior, India, among 1000 school going students found significant association with grades. Higher grade students were more prone to develop sleep quality than lower grade students [22]. Our findings revealed a significant association between satisfaction with academic performance and sleep quality (OR = 1.87, CI: 1.22-2.86) which was supported by the findings from a study conducted in Malaysia [24]. However, no significant association was found in a study conducted in China [21]. This might be due to the difference in the setting and due to difference in sample size.

### 4.3. Behavioral Characteristics and Sleep Quality

The recent study revealed that behavioral characteristics such as alcohol consumption, physical activity, and participation in extracurricular activities were not statistically significant with sleep quality but use of tobacco was significantly associated with sleep quality; however, Bhandari et al. in their study showed no significant association with tobacco use while the alcohol users were good sleepers than nonusers [17]. This might be due to differences in age of the students involved in study.

### 4.4. Psychological Characteristics and Sleep Quality

This study explained that either having girlfriend and boyfriend, feeling lonely, or trying to hurt themselves were found to be statistically insignificant with sleep quality but in a contrary study conducted in China, feeling lonely and ever tried to hurt themselves were significantly associated with sleep quality [21]. Similar to our research findings, the relationship with a teacher and friend (OR = 2.90, CI: 1.80-4.71 and AOR = 2.12, CI: 1.13-4.00, respectively) was found to be statically significant with sleep quality. Those students who had poor relationship with teachers and friends were more likely to develop risks of sleep quality compared to those students who had good relationship [21]. Furthermore, a prior study also demonstrated that running away from home without informing the parents were at risk for sleep problems (*AOR* = 3.15, CI: 1.44-7.21), and our findings explained that those students who frequently shared their feelings and thoughts were less likely to have poor sleep compared to the students who did not share their thoughts to their family [21].

Having a smart phone and regular use of internet were not statistically significant with sleep quality whereas time spend on internet per hour daily and use of internet before falling asleep were found to be associated with sleep quality (OR = 2.10, CI: 1.47-3.10 and AOR = 2.67, CI: 1.61-4.48, respectively). A study conducted in Turkey also showed significant association between poor sleep quality and use of internet per day. Poor sleep quality increased by 2.10 times for an hour spent on the internet [26]. The findings could be attributed to the fact that those students using internet for more than an hour before falling asleep could shorten their sleeping duration leading to poor sleep quality.

### 4.5. Sleep Quality and Depression

Sleep quality was statistically significant with depression among adolescent students in this result. Evidence from other studies clearly shows that sleep quality and depression are strongly interrelated. Those students who have sleep quality have a strong effect on risk of developing depression [3, 27–31]. According to a study conducted by Robert et al. in Texas, risk of developing depression almost increases by fivefolds in a student with sleep quality [3]. In this study, those students lacking proper sleep or having sleep quality were fifteen times more likely to have depression than those students with normal sleep (OR = 15.041, CI: 8.397-26.939). Similar findings were reported in Brazil where the odds of developing risk of depression increases by ten times among poor sleepers (OR = 10.440, CI: 1.400-46.070) [30]. The reason may be that long lasting poor sleep quality may lead to depression among the adolescents, and the poor sleep quality may be due to other factors such as educational, behavioral, and psychological factors including family responsibilities.

This study itself is the first of its kind in countries with population that has unique ethnicity and religion. Although the findings from the small populations cannot be generalizable, but other studies done in larger samples also showed similar results; thus, we believe that the findings can be transferred to the general population of similar settings. There is always more room for new descriptive studies, especially when dealing with such an important subject that has heavy economic, social, and health worldwide implications. Nevertheless, there is a greater need for more interventional research studies that will bring us closer to reducing the rates of poor sleep among adolescents.

## 5. Conclusion

This study concluded that ethnicity, religion, place of residence, drinking status of father, reason for selecting the currently studying faculty, satisfaction with academic performance, use of tobacco, relationship with friends or classmates, more use of internet per day, and use of internet before falling asleep were found to be statistically significant with sleep quality.

## Figures and Tables

**Table 1:** Demographic and economic characteristics of respondents and their family (*n* = 514).

Characteristics	*n* (%)
Age^∗^	
≤17	275 (53.5)
>17	239 (46.5)
Age mean age ± SD (years) 17.4 ± 0.9	
Gender	
Male	270 (52.5)
Ethnicity^∗^	
Brahmin/Chhetri	302 (58.8)
Religion^∗^	
Hindu	461 (89.7)
Place of residence^∗^	
Urban	401 (78.0)
Type of family	
Nuclear	338 (65.8)
Family size	
>5	321 (62.5)
Mean ± SD5.51 ± 2.12	
Parent marital status	
Married	471 (91.6)
Monthly income of family(in NRs)	
≤25000	301 (58.6)
>25000	213 (41.4)
Educational status of father	
≤Secondary	354 (68.9)
>Secondary	160 (31.1)
Education status of mother	
≤Secondary	422 (82.1)
>Secondary	92 (17.9)
Occupational status of father	
Agriculture	202 (39.3)
Business/job	275 (53.5)
Other^#^	37 (7.2)
Occupational status of mother	
Agriculture	291 (56.6)
Business/job	211 (41.1)
Other ^##^	12 (2.3)
Drinking habit of father^∗∗^	
Yes	195 (37.9)
Drinking habit of mother	
Yes	25 (4.9)

^#^Includes foreign employment and labour work, ^##^includes housewife, labour work, and foreign job. ∗∗∗, ∗∗, ∗Significant at <0.000, 0.01, and 0.05, respectively.

**Table 2:** Educational, behavioral, and psychological characteristics of the respondents (*n* = 514).

Characteristics	*n* (%)
Educational	
Grade	
11	287 (55.8)
12	227 (44.2)
Type of school	
Public	252 (49.1)
Private	262 (50.9)
Faculty of the respondent	
Science	149 (28.9)
Management	211 (41.1)
Education	154 (30.0)
Reason for selecting the currently studying faculty^∗∗∗^	
Own decision	413 (80.4)
Others^#^	101 (19.6)
Achievement in last exam	
Pass	402 (78.2)
Fail	110 (21.4)
Satisfied with academic performance^∗^	
Yes	332 (64.6)
No	182 (35.4)
Behavioral	
Tobacco users^∗^	
No	479 (93.2)
Alcohol users	
No	482 (93.8)
Physical exercise	
Regularly	62 (12.1)
Frequently	51 (9.9)
Occasionally	329 (64.0)
Rarely	44 (8.6)
Never	28 (5.4)
Participate in extracurricular activities	
Yes	209 (56.3)
Time spend on internet (hours)^∗∗∗^	
≤ 2	266 (51.8)
> 2	248 (48.2)
Spend time on internet(hours) before going bed^∗∗∗^	
≤ 1	171 (33.3)
> 1	343 (66.7)
Psychological	
Conflict in family	
Yes	406 (79.0)
Family members scold you^∗∗∗^	
No	478 (93.0)
Relationship with friends^∗∗∗^	
Good	253 (49.2)
Average	178 (34.6)
Poor	83 (16.2)
Relationship with teachers^∗∗∗^	
Good	166 (32.3)
Average	220 (42.8)
Poor	128 (24.9)
Have girlfriend/boyfriend	
No	366 (71.2)
Felt lonely	
No	363 (70.6)
Run away from home(ever in their life)^∗∗∗^	
No	473 (92.0)
Hurt own self	
No	395 (76.8)
Sharing with parents	
Frequently	107 (20.8)
Occasionally	361 (70.2)
Rarely	31 (6.1)
Never	15 (2.9)
Sharing with friends	
Frequently	209 (40.7)
Occasionally	248 (48.2)
Rarely	43 (8.4)
Never	14 (2.7)

^#^Includes failing to qualify in other faculty, family pressure, friends pressure. ∗∗∗, ∗∗, ∗Significant at <0.000, 0.01, and 0.05, respectively.

**Table 3:** Multivariate analysis of factors associated with sleep quality.

Characteristics	COR (95% CI)^#^	AOR (95% CI)^##^
Age (≤17 years)	1.59 (1.12-2.27)^∗^	1.55 (1.03-2.33)^∗^
Ethnicity (Brahmin/Chhetri)	1.44 (1.01-2.07)^∗^	1.27 (0.80-2.02)
Religion (Hindu)	2.11 (1.19-3.79)^∗^	1.46 (0.72-2.95)
Residence (urban)	1.64 (1.08-2.51)^∗^	1.53 (0.92-2.52)
Tobacco user (no)	2.38 (1.19-4.90)^∗^	0.98 (0.40-2.39)
Drinking status of father (yes)	1.72 (1.20-2.48)^∗^	1.18 (0.77-1.81)
Reasons of selecting current study (own decision)	1.54 (1.64-3.98)^∗∗∗^	1.77 (1.05-3.00)^∗^
Satisfied with academic performance (yes)	1.87 (1.22-2.86)^∗^	1.44 (0.86-2.40)
Family members scold you (no)	5.01 (2.39-11.51)^∗∗∗^	2.13 (0.90-5.38)
Relationship with friends (good)		
Average	1.15 (0.77-1.72)	1.01 (0.63-1.63)
Poor	3.20 (1.92-5.40)^∗∗∗^	2.12 (1.13-4.00)^∗^
Relationship with teachers (good)		
Average	1.37 (0.89-2.10)	1.07 (0.64-1.78)
Poor	2.90 (1.80-4.71)^∗∗∗^	1.24 (0.66-2.29)
Run away from home **(**no)	4.40 (2.06-8.42)^∗∗∗^	3.15 (1.44-7.21)^∗∗^
Hours spent on internet(≤ 2)	2.10 (1.47-3.10)^∗∗∗^	1.07 (0.66-1.72)
Hours spend on internet before going to bed(≤ 1)	3.27 (2.24-4.81)^∗∗∗^	2.67 (1.61-4.48)^∗∗∗^

(): reference category. ∗∗∗, ∗∗, ∗Significant at <0.000, 0.01, and 0.05, respectively; #COR calculated from the bivariate logistic regression; ^##^AOR calculated from the multivariate logistic regression. Note: if, in the bivariate analysis, the variable was insignificant, it was not included in the multivariate analysis. Fitting null model for pseudo-*R*^2^ (pseudo-*R*^2^ was calculated by using the “Pscl” package). IIH: log‐likelihood from the fitted model = −296.14. IIhNull: the log‐likelihood from the intercept only restricted = −344.25. G2: minus two times the difference in the log‐likelihoods = 96.23. McFadden: McFadden′s pseudo − *R*^2^ = 0.14. *R*^2^ML: maximum likelihood pseudo − *R*^2^ = 0.17. *R*^2^CU: Cragg and Uhler′s pseudo − *R*^2^ = 0.23. The multicollinearity was assessed by using VIF (variance infection factor) by using “car” package; all the VIF were less than 5. So, there is no multicollinearity in this model.

## Data Availability

The datasets and materials used and/or analyzed during the current study can be made available from the corresponding author on reasonable request.
